# Prevalence of Obesity and Lifestyle Risk Factors Following Two Years’ COVID-19 Related Service Closure at Wellness Center, Primary Health Care

**DOI:** 10.2147/DMSO.S433978

**Published:** 2023-11-29

**Authors:** Sarah Musa, Ayman Al-Dahshan, Rajvir Singh

**Affiliations:** 1 Department of Preventative Health, Primary Health Care Corporation, Doha, Qatar; 2 Department of Medical Education, Hamad Medical Corporation, Doha, Qatar; 3 Department of Adult Cardiology, Heart Hospital Hamad Medical Corporation, Doha, Qatar

**Keywords:** COVID-19, lockdown, lifestyle behavior, physical activity, obesity, stress, sleep

## Abstract

**Background:**

COVID-19 lockdown has affected health behaviors and daily life in unprecedented ways. This study aimed to assess (i) the prevalence of lifestyle behaviors including physical activity, sedentary behavior, sleep, and stress levels during the COVID confinement, and (ii) evaluate anthropometric measures, body composition, cardiopulmonary and muscular endurance among regular Wellness Center attendees, following two years’ service closure.

**Methods:**

A cross-sectional study was undertaken between 1^st^ June 2022 and 30^th^ January 2023. A structured validated questionnaire was utilized to retrospectively collect data related to the closure period, and quantitative objective measurements were obtained at the time of data collection.

**Results:**

A total of 100 adults with a mean age of 49.6 ± 10.5 years, mostly female (80%) and Qatari (76%) participated in the study. The results revealed high rates of low physical activity (47%), sedentary behavior (62%), poor sleep (58%), and stress levels (61% moderate and 5% high). Results also confirmed high prevalence of obesity (57%) marked by BMI and body circumferences, fat mass (34 ± 10.5 kg)/fat percentage (42.7 ± 7.2%) and muscle mass (20.6 ± 5.4 kg)/muscle percentage (25.4 ± 3.9%) above and below normal levels, respectively. The average cardiopulmonary (mean VO2 MAX was 15.5 ± 11 mL O_2_kg^−1^min^−1^) and muscular endurance (pushups per min 19 ± 8.9, L-sit 46 ± 35 secs, plank 42.7 ± 27.8 secs) were distinctly below normal levels for age and sex.

**Conclusion:**

The findings indicate that physically active adults have experienced adverse lifestyle behaviors during the COVID-19 lockdown. Obesity, unfavorable body composition, low cardiopulmonary and muscular endurance were evident. More emphasis should be put on the development of targeted intervention strategies to promote positive lifestyle behaviors during any potential future crises.

## Introduction

The novel coronavirus (COVID-19) was declared a pandemic and a public health emergency by the World Health Organization (WHO) in March 2020.^1^ Public health authorities around the globe have undertaken stringent containment measures including lockdowns, quarantine, social distancing, travel restriction and schools’ closure.^2^,^3^ Prolonged lockdown, especially within the high-risk environments (ie, fitness facilities), has compromised the ability to maintain physical activity (PA) and healthy lifestyles.^4^ A 27% reduction in daily steps was observed only within 30 days of the pandemic declaration^5^ in 450,000 individuals around 187 countries. Reductions in all levels of PA and a third increase of daily sitting as well as unhealthy eating patterns were observed.^6^ Data from the Active Lives Adult Survey show that during the initial phase of the pandemic, between mid-November 2019 and mid-November 2020, 39% of adults aged 16 year and above were engaged in physical activity below 150 minutes per week.^7^ The pandemic has also enhanced the risk factors associated with poor mental health, including social isolation, financial uncertainties, and health concerns, which led to increased stress, anxiety, depression, and sleep disorders.^8^,^9^

According to the WHO guidelines, adults aged 18 to 64 years are recommended to engage in at least 150 minutes of moderate-intensity PA or 75 minutes of vigorous-intensity PA each week or an equivalent combination of moderate-to-vigorous PA (MVPA).^10^ Regular PA has profound positive effects on both physical and mental well-being, reducing all-cause mortality and risk factors such as obesity, diabetes mellitus, and cardiovascular diseases.^11^,^12^ PA may improve insulin sensitivity, glycemic control, and lipoprotein profile among individuals with type 2 diabetes.^13^ A meta-analysis indicated that 8 weeks or more of PA had successfully lowered the level of glycated hemoglobin (HbA1C) from 8.31% to 7.65%, independent of body mass.^14^ PA was also linked to risk reduction of COVID-19 infection, hospitalization, mortality, and severe illness, by 11%, 36%, 43% and 44%, respectively.^15^

People were advised to continue safe and simple strategies to maintain health-related physical fitness during the pandemic. Home-based exercises using training equipment, such as a treadmill, a cycle ergometer, a rowing machine, and dumbbells/resistance bands in conjunction with walking/jogging either inside or outside the house if able to maintain physical distancing, were deemed alternatives.^16^,^17^ Active video games were also found to increase the energy expenditure comparable to the moderate intensity walking and might be considered an option, especially among children and adolescents.^18^

In Qatar, six Wellness Centers have been established across primary health care aiming to provide a 12-week weight management program, exercise regimens, and behavioral modification counseling by healthy lifestyle experts. However, these services were suspended from the start of the pandemic (March 2020) and since then, there has been no contact with wellness ‎clients. In July 2021, wellness services were resumed at five out of six health centers apart from Rawdat Al-Khail Health Center (RAK-HC) which was dedicated as a COVID-19 facility and resumed its services only on March 6^th^, 2022. The unique experiences of wellness clients, their health issues, and the ways in which they have dealt with routine change and lifestyle-related behaviors during the pandemic have remained underexplored. Therefore, the current study aimed to assess (i) the prevalence of lifestyle behaviors including physical activity, sedentary behavior, sleep, and stress levels, and (ii) evaluate different anthropometric measures, body composition, cardiopulmonary and muscular endurance among regular Wellness Center attendees, following two years’ service closure.

## Materials and Methods

### Study Design and Participants

This cross-sectional study was conducted at Wellness Center in RAK-HC between 1^st^ June 2022 to 30^th^ January 2023, two years after service closure. Participants were invited to take part after explaining the study objectives. The inclusion criteria were as follows: age ≥18 years and previously active wellness client who met the physical activity recommendations of at least 150–300 minutes of moderately intensive physical activity (equivalent to 3.0–5.9 Metabolic Equivalent of Task, METs) or 75 minutes vigorous activity (6.0 and higher METs), per week or an equivalent combination of both, prior to service closure. The exclusion criteria were newly joined clients or who were absent for 2–3 consecutive weeks prior to the closure. A convenience sample comprised 100 participants of both sexes (20 males and 80 females) took part in this study. According to 2018–2022 data, the ratio of male:female at Wellness Center is 1:4 with 80% above the age of 37 years. The study protocol was approved by the Institutional Review Board (IRB) of the Primary Health Care Corporation under the reference number (PHCC/DCR/2022/03/012). Written, informed consent was obtained from all the participants. Participants could withdraw or decline participation at any time during the study.

### Data Collection

Eligible participants have completed a questionnaire upon their re-joining of wellness services. The questionnaire comprised two sections, namely (i) interview-based questionnaire to collect data on sociodemographic, clinical characteristics, physical activity, sedentary behavior, and sleep habit and (ii) self-administered questionnaire to assess stress level. Data related to anthropometric measurements, body composition, cardiopulmonary and muscular endurance were collected at baseline.

### Study Variables

The sociodemographic data included information on age, sex, nationality, education level, occupation, and monthly income. Clinical data were recorded as the presence or absence of comorbidities including diabetes mellitus, dyslipidemia, thyroid disease, hypertension, cardiac, asthma and others.

#### Physical Activity and Sedentary Behavior

The measure of PA level was assessed using the International Physical Activity Questionnaire Short Form (IPAQ-SF) that has good reliability and validity after being tested in many countries.^19^ The tool assesses the types of PA intensity and time spent physically active as well as sitting time that people do as part of their daily lives to estimate the total PA in (METs)-minutes/week and time spent sitting/week. Later, PA was categorized further into low, moderate, or high based on the calculated METs per week as the following:

- Low-intensity PA: participants who do not meet the 600 METs min/week criteria for moderate or vigorous PA.
- Moderate-intensity PA: participants who satisfy any of the following requirements: (a) three or more days of vigorous activity of at least 20 minutes/day or (b) five or more days of moderate intensity activity or walking of at least 30 minutes/day or (c) five or more days of any combination of walking, moderate intensity or vigorous intensity activities achieving a minimum of at least 600 METs-minutes/week.
- High-intensity PA: participants must satisfy the following criteria: (a) three or more days of intense activity totaling at least 1500 METs-minutes/week or (b) seven or more days of any combination of intense, moderate, or walking activity totaling at least 3000 METs-minutes/week.

Self-reported PA, weight changes, sedentary behavior (SB) and screen time (ST) during the COVID-19 wellness service closure were collected as well. Sitting time in hours was calculated as mean ± SD.

#### Sleep Assessment

Participants reported the hours spent sleeping per day, while sleep quality was assessed using yes/no questions to evaluate habitual sleep pattern and sleep problems as the following: 1. self-evaluation of sleep quality (poor/good/very good) 2. sleep badly and restlessly, 3. hard to go to sleep within 30 mins and 4. wake up early and not been able to get back to sleep.

#### Stress Assessment

The Perceived Stress Scale (PSS-10)^20^ was used to assess the overall stress level. The tool was designed to assess the degree of stress people felt in unpredictable, out-of-control, and overloaded situations. It comprised of ten questions about feelings and thoughts during the closure time. Each item was rated on a 5-point scale from never (scored zero) to very often (scored four). The tool classified participants according to their stress level into low (0–13), moderate (14–26), or high (27–40).

#### Anthropometric Variables

The weight in kilograms (kg) was measured using SECA scales with an accuracy of 0.1 kg. Height in centimeters (cm) was measured using the SECA stadiometer to an accuracy of 1 millimeter. Body mass index (BMI) was calculated as kg/m^2^. Based on the definition of the WHO,^21^ we categorized BMI as underweight (BMI < 18.5 kg/m^2^), normal weight (BMI = 18.5–24.9 kg/m^2^), pre-obesity (BMI = 25–29.9 kg/m^2^), obesity class I (BMI 30.0–34.9 kg/m^2^), obesity class II (35.0–39.9 kg/m^2^) and obesity class III (BMI≥ 40 kg/m^2^). Circumferences were measured using a tape measure and standard protocol according to the American College of Sports Medicine (ACSM) guidelines,^22^ as the following:

- Waist circumference (WC) in centimeters: with the client standing upright and relaxed, a horizontal measure was taken at the narrowest part between the umbilicus and the xiphoid process of the torso.
- Mid-arm circumference (MUAC) in centimeters: with the client standing erect and arms hanging freely at the sides, with hands facing the thigh, a horizontal measure was taken midway between the acromion and olecranon processes.
- Calf circumference (CalF) in centimeters: with the client standing (feet apart 20 cm), a horizontal measure was taken at the level of the maximum circumference between the knee and the ankle, perpendicular to the long axis.
- Thigh circumference (TC) in centimeters: with legs slightly apart, the maximal girth of thigh below the gluteal fold is measured.
- Chest circumference (CC) in centimeters: measured at the level of the nipple, at the end of expiration, to the nearest 0.1 cm.
- Waist-to-hip-ratio (WHR): calculated as the circumference of the waist divided by the circumference of the hip.

All circumferences were measured with an accuracy of one millimeter by a standard tape soft meter.

#### Body Composition Analysis

*A* medical body composition analyzer (SECA mBCA 515/514 scale)^23^ using bioelectrical impedance analysis (BIA) was used to determine fat mass (FM) in kg, percentage fat mass (PFM), muscle mass (MM) in kg, percentage muscle mass (PMM) and Total Body Water (TBW) percentage. Participants were asked to remove any outer clothing, both shoes/socks or tights, empty pockets, and chunky jewelry. They were asked to stand on the SECA machine with their heels, and balls of each foot are touching the metal electrodes on the base of the machine. Hands are positioned on either side of the machine to be in contact with the electrodes. All measurements were performed by the same trained personnel based on the same protocol.

*2.3.6. Cardiorespiratory fitness (CRF*): Measured using VO2 MAX (mL of O_2_/kg of body weight/min using cooper test). VO2 MAX is the maximum rate of consumption of oxygen per minute during maximal aerobic exercise and may be measured directly by collecting expired gases in a controlled laboratory setting or indirectly through field tests such as Cooper test. Cooper 12-minute test was used to cover the greatest distance in the allotted period either walking, running on a treadmill or cycling using leg ergometry. VO2 MAX was estimated using the following ACSM metabolic equations:^22^

$${\mathrm{Walking\ VO2\ MAX=}}\left({{{\mathrm{S}}^{\mathrm{a}}}{\mathrm{x0}}{\mathrm{.1}}}\right){\mathrm{+}}\left({{\mathrm{Sx}}{{\mathrm{G}}^{\mathrm{b}}}{\mathrm{x1}}{\mathrm{.8}}}\right){\mathrm{+3}}{\mathrm{.5}}$$

$${\mathrm{Running\ VO2\ MAX=}}\left({{{\mathrm{S}}^{\mathrm{a}}}{\mathrm{x0}}{\mathrm{.2}}}\right){\mathrm{+}}\left({{\mathrm{Sx}}{{\mathrm{G}}^{\mathrm{b}}}{\mathrm{x0}}{\mathrm{.9}}}\right){\mathrm{+3}}{\mathrm{.5}}$$

$${\mathrm{Leg\ ergometry\ VO2\ MOX=}}\left({{{\mathrm{W}}^{\mathrm{c}}}{\mathrm{/}}{{\mathrm{M}}^{\mathrm{d}}}{\mathrm{x10}}{\mathrm{.8}}} \right){\mathrm{+3}}{\mathrm{.5}}$$

S^a^= speed of treadmill in m·min-1; 1 mph = 26.8 m·min-1.

G^b^= grade (% incline) of treadmill in decimal.

W^c^= power output in watts; 1 W = 6 kgm·min-1.

M^d^= body mass in kilograms; 1 kg = 2.2 lb.

*2.3.7. Muscular endurance*: Measured by “push-up”, “L-sit” and “plank” tests according to ACSLM guidelines.^21^ This data was obtained by the gym instructors during the initial assessment using validated tools of measures.

- Push-up: measures the upper body strength and endurance. The test was performed starting from the prone position, with elbows extended and palms of the hand resting on the floor at chest level, while the trunk, thighs, and legs must be aligned throughout the execution. Participants bent the elbow symmetrically until reaching a lower angle of 90 degrees between the arm and forearm. When flexing the elbows, the participant lowered the body and pushed back up by extending the elbows. The number of push-ups was counted in 1 minute.
- L-sit: measures static leg endurance. Participants feet flat on floor, and shoulder width apart, knees at 90-degree angle with the shoulder against the wall, and arms hanging straight down. Failure was defined as 5-degree deviation from a 90-degree knee angle. Participants were instructed to look straight ahead throughout the duration of the trial. Time in seconds was recorded using a stopwatch.
- Plank: isometric exercise measures muscular endurance of the abdominal core. Participants maintained a prone position with body weight supported by the toes and forearms. They were instructed to maintain a neutral position of the spine and pelvis and to breathe normally. A stopwatch was used to record the holding time. The test was terminated when participants were unable to maintain their posture, or their pelvis moved up or down five or more cm.

### Data Analysis

Mean and standard deviation (mean ±SD) were calculated for continuous variables whereas frequency distribution with percentages were calculated for categorical variables. Graphs were presented to demonstrate stress level and distribution of the subjects according to BMI levels. Student’s *t*-test was used to analyze body measurements/compositions and cardiopulmonary/metabolic endurance according to sex. Chi-square tests were applied to examine the association between sociodemographic/clinical characteristics and physical activity level as well for WC/WHR according to sex. One way ANOVA was performed to assess the mean differences between anthropometric/body composition and physical activity level and to analyze cardiopulmonary/muscular endurance according to age groups. The P value of ≤0.05 (two tailed) was considered as statistically significant level. SPSS 29.0 statistical package was used for the analysis.

## Results

### Sociodemographic Characteristics of Participants

Table 1 presents the sociodemographic and clinical characteristics of the study population. Of the 100 participants with a mean age of 49.6 ± 10.5, the majority were females (80%) and Qataris (76%). Most of the participants were university-educated (72%) and over half were employed (58%), of which 38% were in an office job and 15% were in the healthcare sector, whereas 42% were unemployed. Most of the participants (70%) were married, and 16% were single. Chronic diseases among participants included diabetes mellitus (15%), dyslipidemia (13%), hypothyroidism (13%), hypertension (12%), cardiac disease (3%) and asthma (1%). (Table 1)

**Table 1:** Sociodemographic and Clinical Characteristics of Participants (n = 100)

Characteristic	Number	Percentage (%)
**Age (years)**	100	
Mean ± SD	49.6±10.5	
**Gender**		
Male	20	20%
Female	80	80%
**Education level**		
Illiterate	0	0%
Primary	0	0%
Preparatory	9	9%
Secondary	19	19%
University or higher	72	72%
**Occupation**		
Unemployed/resigned	42	42%
Student	04	04%
Office job	38	38%
Professional	01	01%
Healthcare	15	15%
**Nationality**		
Qatari	76	76%
Non-Qatari	24	24%
**Marital status**		
Single	16	16%
Married	70	70%
Divorced	10	10%
Widow	4	4%
**Monthly income (QR)**		
<5000	10	10%
5000-<10,000	17	17%
10,000-<20,000	19	19%
20,000 −30,000	20	20%
>30,000	34	34%
**Chronic disease**		
Diabetes Mellitus	15	15%
Dyslipidemia	13	13%
Hypothyroidism	13	13%
Hypertension	12	12%
Cardiac disease	3	3%
Asthma	01	01%

**Abbreviations**: SD, standard deviation; QR, Qatari Riyals.

### Physical Activity and Sedentary Behavior

Table 2 provides data on the PA level, self-reported weight change and SB during service closure. The IPAQ short form was used to examine PA levels, which were divided into three categories: low, moderate, and vigorous PA, with 47%, 47%, and 6% of the sample population fitting into each of those categories, respectively. The mean METs-minutes/week was 1038 ± 1172 minutes per week, and the mean sitting time was 5.1 ± 4.7 hours/day. The majority (80%) of the participants had either never (28%) or occasionally (52%) engaged in PA during the time-of-service closure, with only 20% could maintain regular PA. The most reported reasons for not engaging in PA were routine disruption (49%), lack of supportive environment (17%) and lethargy (14%). Self-reported weight change indicates that 54% of the participants have gained weight, while 31% had no change and 15% had lost weight. More than half of the participants (62%) were sedentary for more than 8 hours a day, and the mean ST was 4 ± 2.8 hours/day (Table 2).

**Table 2:** Physical Activity Level and Sedentary Behavior During the COVID-19 Related Wellness Service Closure (n = 100)

Variable	Number	Percentage (%)
PA level (IPAQ)		
Low	47	47%
Moderate	47	47%
High	06	06%
METs (Minutes/week) Mean ± SD	1038±1172	
Engaged in PA		
Never	28	28%
Occasionally	52	52%
Regularly	20	20%
Reasons not engaging in PA (n = 80)		
Lethargy	14	14%
Disrupted routine	49	49%
Lack of support	17	17%
SB > 8 hours/day		
No	62	62%
Yes	38	38%
ST (hrs.) Mean ± SD	4 ± 2.8	
Weight change		
No change	31	31%
Lost weight	15	15%
Gained weight	54	54%

**Abbreviations**: SD, standard deviation; IPAQ, International Physical Activity Questionnaire; PA, physical activity; SB, sedentary behavior; ST, screen time; METs, Metabolic Equivalent of Task.

### Sleep Quality

Table 3 presents the sleep habits during the period of service closure. The average self-reported sleep quality was described as good in 59% participants and very good in 19%, while 22% reported poor sleep quality. Furthermore, 41% of participants slept badly and restlessly, 55% felt difficulty sleeping within 30 minutes, and 53% woke up early and had difficulty going back to sleep. More than half of the participants (58%) had sleep deprivation and slept less than 7 hours, 39% were able to maintain sleep duration at 7–9 hours, while only 3% slept more than 9 hours daily (Table 3).

**Table 3:** Sleep Status During the COVID-19 Related Wellness Services Closure (n = 100)

Variable	Number	Percentage (%)
Sleep quality		
Poor	22	22%
Good	59	59%
Very good	19	19%
Slept badly and restlessly		
No	59	59%
Yes	41	41%
Difficulty sleep within 30 minutes		
No	45	45%
Yes	55	55%
Woken up early/unable to go back to sleep		
No	47	47%
Yes	53	53%
Sleep duration (hours)		
< 7	58	58%
7–9	39	39%
>9	03	3%

### Perceived Stress Level

**Figure 1:**
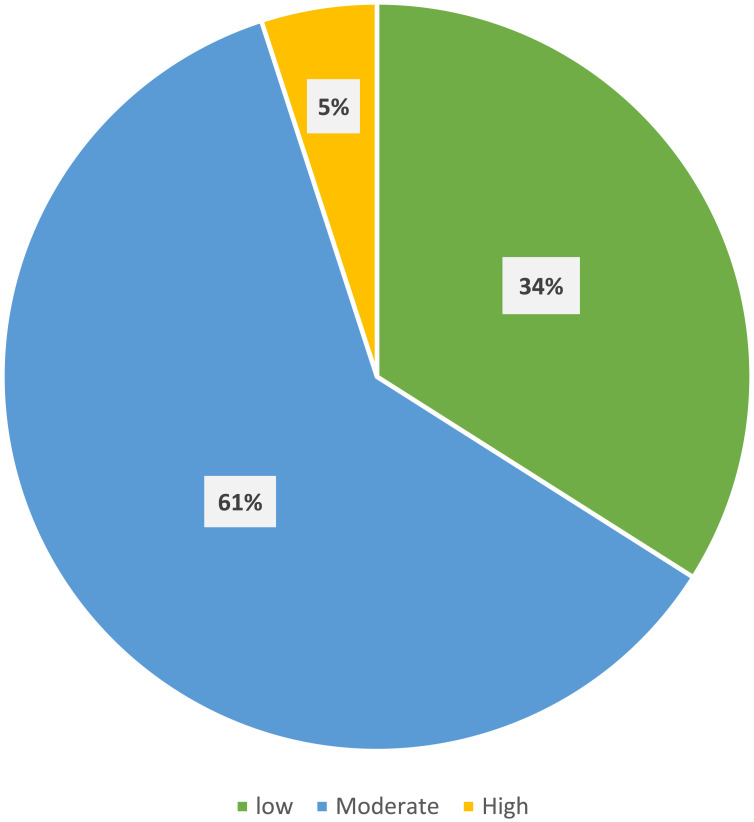
Stress level based on the perceived stress scale during COVID-related wellness service closure (n = 100).

Figure 1 presents the stress level based on the PSS-10 scale among study participants. Respondents reported an average score of 16.1 ± 6.5 for perceived stress, with 61% experiencing moderate and 5% high stress levels (Figure 1).

### Body Measurements and Composition

**Figure 2:**
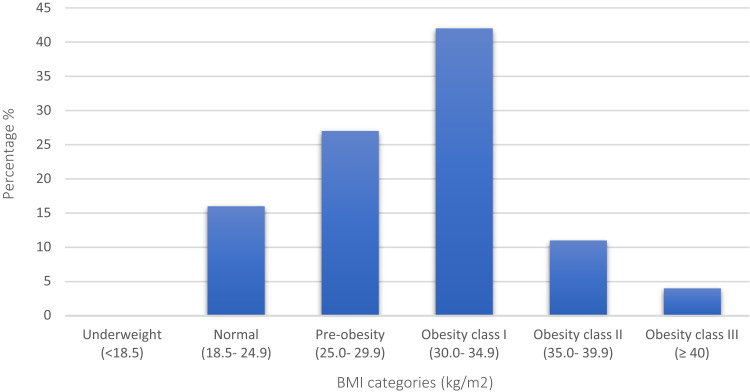
BMI categories based on the WHO guidelines (n = 100).

Table 4 shows descriptive statistics of body measurements and composition according to sex. The mean height of the participants was 160 ± 16 cm, and the mean weight was 79 ± 15 kg with a mean BMI of 30.5 ± 5 kg/m^2^. As per the BMI categories, more than half of the population (57%) were in the obesity category (BMI ≥ 30 kg/m^2^); 42% in obesity class I, 11% as obesity class II and 4% as obesity class III, followed by 27% participants in pre-obesity category (BMI 25.0–29.9 kg/m^2^) and only 16% were falling under the normal BMI category (BMI 18.4–24.9 kg/m^2^) (Figure 2). The mean right and left MUAC were 33 ± 3.9 cm and 32.5 ± 3.9 cm, CC was 103 ± 9.1 cm, and WC was 102 ± 12.5 cm. The mean WHR was 0.92 ± 0.10. The participants were with a mean right CalF of 39 ± 4.2 cm and left CalF of 39 ± 4.2 cm, right TC of 61 ± 9 cm, left TC of 60.9 ± 8.9 cm, and a RHR of 76.9 ± 9.3 bpm. When analyzing their body composition, the mean FM and PFM were 34 ± 10.5 kg and 42.7 ± 7.2%, while the mean MM and PMM were 20.6 ± 5.4 kg and 25.4 ± 3.9%, respectively. The mean TBW percentage was 40.6 ± 6.4%. Males had significantly higher WC (p = 0.04), WHR (p = 0.001), MM & PMM (p = 0.01), and TBW (p = 0.01) than females; however, TC, FM, and PFM were higher among females (p = 0.01) (Table 4).

**Table 4:** Body Measurements and Composition According to Sex (n = 100)

Variable	Male Mean ± SD	Female Mean ± SD	Total Mean ± SD	P value
Height (cm)	169.2 ± 6.8	157.6 ± 14.8	160 ± 16	0.17
Weight (kg)	85.7 ± 13.1	77.4 ± 14.8	79 ± 15	0.03*
BMI (kg/m^2^)	29.8 ± 3.8	31.0 ± 5.3	30.5 ± 5	0.49
Right MUAC (cm)	31.5 ± 2.6	32.9 ± 4.1	33 ± 3.9	0.15
Left MUAC (cm)	31.4 ± 2.6	32.9 ± 4.1	32.5 ± 3.9	0.11
CC (cm)	105 ± 7.0	102.3 ± 9.6	103 ± 9.1	0.24
WC (cm)	106 ± 8.1	101 ± 13	102 ± 12.5	0.04*
WHR	0.98 ± 0.05	0.91 ± 0.10	0.92 ± 0.10	0.001*
Right *CalF* (cm)	39.3 ± 4.2	39.2 ± 4.2	39 ± 4.2	0.001*
Left *CalF* (cm)	38.9 ± 4.3	39.2 ± 4.2	39 ± 4.2	0.001*
Right TC (cm)	55.1 ± 11.0	63.0 ± 7.7	61 ± 9	0.001*
Left TC (cm)	54.8 ± 11.0	62.5 ± 7.6	60.9 ± 8.9	0.001*
RHR (b.p.m)	68.9 ± 4.9	78.9 ± 9.1	76.9 ± 9.3	0.001*
FM (kg)	29.9 ± 8.6	34.9 ± 10.7	34 ± 10.5	0.001*
PFM (%)	34.1 ± 4.9	44.9 ± 5.9	42.7 ± 7.2	0.001*
MM (kg)	26.5 ± 3.4	19.1 ± 4.8	20.6 ± 5.4	0.001*
PMM (%)	31.0 ± 2.6	24.1 ± 2.7	25.4 ± 3.9	0.001*
TBW (%)	46.8 ± 3.9	39.0 ± 5.9	40.6 ± 6.4	0.001*

**Note**: *P value ≤ 0.05 indicates statistically significant difference between sexes by the Student *t* test.

**Abbreviations**: BMI, body mass index; MUAC, mid-upper arm circumference; CC, chest circumference; WC, waist circumference; WHR, waist hip ratio; CalF, calf circumference; TC, thigh circumference; RHR, resting heart rate; b.p.m, beats per minute; FM, fat mass; PFM, percentage fat mass; MM, muscle mass; PMM, percentage muscle mass; TBW, total body water.

According to WC, females (83.8%) were more likely to exhibit higher obesity-related and cardiometabolic risk when compared to males (60%) (p = 0.02). All males (100%) and half of the females (50%) were at increased risk of the same when WHR is considered (Table 5).

**Table 5:** Cardiometabolic Risk According to Waist Circumference and Wasit hip Ratio by Sex (n = 100)

Variable	Category	Male, N = 20 (20%)		Female, N = 80 (80%)		P value
WC	Low	<94 cm	–	<80 cm	–	
	Moderate	94–102 cm	8 (40.0)	80–88 cm	13 (16.3)	0.02*
	High	>102 cm	12 (60.0)	>88 cm	67 (83.8)	
WHR	Increased risk	≥0.85	20 (100)	≥0.90	40 (50)	0.001*

**Note**: *P value ≤ 0.05 indicates statistically significant difference by the Chi-square test.

**Abbreviations**: WC, waist circumference; WHR, waist hip ratio.

### Cardiopulmonary and Muscular Fitness

Table 6 presents cardiopulmonary and muscular endurance according to sex. The total mean VO2 MAX was 15.5 ± 11 (mL O_2_kg^−1^min^−1^) with mean METs of 3.2 ± 1.2 (mL/kg). Males had significantly higher VO2 MAX when compared to females (p = 0.01). The mean number of pushups in one minute was 19 ± 8.9, duration of L-sit/wall sits was 46 ± 35 seconds, and durations of planks were 42.7 ± 27.8 seconds. No significant difference was detected between males and females in terms of muscular endurance.

**Table 6:** Cardiopulmonary and Muscular Endurance According to Sex (n = 100)

Variable	Male Mean ± SD	Female Mean ± SD	Total Mean ± SD	P value
VO2 MAX (mL O_2_kg^−1^min^−1^)	21.0±9.2	14.1±11.2	15.5±11.3	0.01*
METs (mL/kg)	4.3±2.2	2.9±0.6	3.2±1.2	0.01*
Push up (no. repetition/min)	21.0±7.9	18.6±9.1	19.1±8.9	0.29
L-sit/wall sit (duration in secs)	46.1±28.0	46.2±36.8	46.2±35.1	0.98
Plank (duration in sec)	49.5±25.5	41.0±28.2	42.7±27.8	0.22

**Note**: *P value ≤ 0.05 indicates statistically significant difference between sexes by the Student *t* test.

**Abbreviations**: VO2 MAX, maximal oxygen consumption; METs, metabolic equivalent of task.

Table 7 presents cardiopulmonary and muscular endurance according to age groups. Participants aged between 35 and 39 had the highest VO2 MAX and METs compared to all other age groups (p = 0.001). However, age was not a significant moderator of muscular endurance.

**Table 7:** Cardiopulmonary and Muscular Endurance According to Age Groups (n = 100)

Variable	(20–29) Mean ± SD	(30–39) Mean ± SD	(40–49) Mean ± SD	(50–59) Mean ± SD	(60–69) Mean ± SD	(70–79) Mean ± SD	P value
VO2 MAX (mL O_2_kg^−1^min^−1^)	11.5±2.3	25.9±21.2	13.4±5.8	12.9±5.3	15.1±6.8	10.3±2.1	0.001*
METs (mL/kg)	2.7±0.5	4.3±1.9	3.0±0.7	3.0±1.0	2.9±0.6	2.7±0.6	0.001*
Push up (no. repetition/1 min)	14.8±8.6	19.5±8.6	20.5±9.9	18.2±8.9	19.5±8.3	24.5±0.7	0.71
L-sit/wall sit (duration in sec)	47.2±55.0	60.9±55.5	48.1±19.8	40.2±29.2	38.9±26.1	52.0±39.6	0.43
Plank (duration in sec)	41.0±24.4	51.2±30.7	45.5±26.7	38.8±29.0	35.9±19.7	57.5±38.9	0.56

**Note**: *P value ≤ 0.05 indicates statistically significant difference for the One-way ANOVA.

**Abbreviations**: VO2 MAX, maximal oxygen consumption; METs, metabolic equivalent of task.

### Physical Activity by Sociodemographic and Clinical Factors

Table 8 demonstrates the sociodemographic and clinical characteristics according to the physical activity level. Females were more likely to exhibit low physical activity level when compared to males (p = 0.003) whereas; no statistically significant association was found with other factors.

**Table 8:** Sociodemographic and Clinical Characteristics According to the Physical Activity Level (n = 100)

Variable	Category	Low PA (47%)	Moderate PA (47%)	High PA (6%)	P value
Gender	Male	3 (6.4)	14 (24.8)	3 (50)	
	Female	44 (93.6)	33 (70.2)	3 (50)	0.003*
Education	Preparatory	4 (8.5)	3 (6.4)	3 (33.3)	0.17
	Secondary	9 (19.1)	8 (17.0)	2 (33.2)	
	University	34 (72.3)	36 (76.6)	2 (33.3)	
Occupation	Unemployed	19 (40.4)	21 (44.7)	2 (33.3)	0.75
	Student	3 (6.4)	1 (2.1)	0 (0)	
	Office job	17 (36.2)	17 (36.2)	4 (66.7)	
	Professional	0 (0)	1 (2.1)	0 (0)	
	Healthcare	8 (17)	7 (14.9)	0 (0)	
PHCC staff	Yes	9 (19.1)	7 (14.9)	1 (16.7)	0.33
Nationality	Qatari	36 (76.6)	34 (72.3)	6 (100)	0.33
Marital status	Single	10 (21.3)	6 (12.8)	0 (0)	0.46
	Married	27 (57.4)	36 (76.6)	6 (100)	
	Divorced	6 (12.8)	4 (8.5)	0 (0)	
	Widow	3 (6.4)	1 (2.1)	0 (0)	
Income in QR	<5000	8 (17)	2 (4.3)	0 (0)	0.16
	5000<10,000	9 (19.1)	7 (14.5)	1 (16.7)	
	10,000<20,000	12 (25.5)	7 (14.9)	0 (0)	
	20,000–30,000	7 (14.9)	11 (23.4)	2 (33.3)	
	>30,000	11 (23.4)	20 (42.6)	3 (50)	
Diabetes Mellitus	Yes	7 (14.9)	7 (14.9)	1 (16.7)	0.99
Dyslipidemia	Yes	3 (6.4)	10 (21.3)	0 (0)	0.06
Hypothyroidism	Yes	7 (14.9)	5 (10.6)	1 (16.7)	0.80
Cardiac disease	Yes	0 (0)	3 (6.4)	0 (0)	0.18
HTN	Yes	2 (4.3%)	9 (19.1)	1 (16.7)	0.08
Asthma	Yes	1 (2.1%)	0 (0)	0 (0)	0.57

**Note**: *P value ≤ 0.05 indicates statistically significant difference by the Chi-square test.

**Abbreviations**: PHCC, Primary Health Care Corporation; QR, Qatari Riyals; DM, diabetes mellitus; HTN, hypertension.

Table 9 illustrates the anthropometric and body composition according to the physical activity level. Higher FM (p = 0.02), higher PFM (p = 0.005), lower PMM (p = 0.01) and lower RHR (p = 0.001) were significantly associated with low physical activity, whereas no mean difference was found among all levels of PA and other variables.

**Table 9:** Anthropometric and Body Composition According to the Physical Activity Level (n = 100)

Variable	Low PA Mean ± SD	Moderate PA Mean ± SD	High PA Mean ± SD	P value
Age	48.0±10	51±10.9	52.2±7.0	0.36
Height (cm)	162±6.2	158±12.6	163±7.6	0.47
Weight (kg)	81±15	76.3±13	83.3±22	0.21
BMI (kg/m^2^)	31.5±4.5	29.4±5.2	31.2±6.0	0.12
Right MUAC (cm)	33.5±3.8	31.5±4.0	33.2±2.6	0.04*
Left MUAC (cm)	33.5±3.7	31.6±4.1	33.2±2.9	0.06
CC (cm)	105±8.8	100.5±9.2	106±8.8	0.06
WC (cm)	104.5±13	99.2±11	105±13.7	0.10
WHR	0.92±0.09	0.92±0.10	1.0±0.11	0.17
Right *CalF* (cm)	39.9±3.8	38.5±4.6	39.5±3.6	0.27
Left *CalF* (cm)	39.9±3.6	38.4±4.5	38.7±3.8	0.23
Right TC (cm)	63.2±7.4	59.7±10.4	61.8±7.7	0.17
Left TC (cm)	62.6±7.2	54.5±10.3	60±8.5	0.22
RHR (b.p.m)	81±9.9	73.2±7.3	75.2±6.8	0.001*
FM (kg)	36.9±10.8	30.8±9.1	35.2±12.8	0.02*
PFM (%)	45.1±6.7	40.4±7.1	41.5±6.9	0.005*
MM (kg)	20.6±5.8	20.3±5.0	22.0±6.6	0.77
PMM (%)	24.3±3.1	26.5±4.2	26.8±4.1	0.01*
TBW (%)	39.7±4.9	41.8±6.8	36.8±11.2	0.10

**Note**: *P value ≤ 0.05 indicates statistically significant difference for the One-way ANOVA.

**Abbreviations**: PA, physical activity; BMI, body mass index; MUAC, mid-upper arm circumference; CC, chest circumference; WC, waist circumference; WHR, waist hip ratio; CalF, calf circumference; TC, thigh circumference; RHR, resting heart rate; b.p.m, beat per minutes; FM, fat mass; PFM, percentage fat mass; MM, muscle mass; PMM, percentage muscle mass; TBW, total body water.

## Discussion

In the present study, we provided for the first-time data on the prevalence of PA and various lifestyle behaviors among physically active patients, throughout the period of two years during the pandemic and service closure. In a sample of previously regular Wellness Center attendees who were largely females (80%) of Qatari nationality and mean age of 49.6 ±10.5 years, both negative and positive impacts were observed; however, unfavorable changes were more commonly witnessed, including high percentages of physical inactivity, SB, obesity, poor sleep, increased stress level, and low cardiopulmonary and muscular fitness. Despite the recommendations that COVID-19 preventive measures should not hinder people from being physically active, the current findings demonstrate insufficient PA in about half of our participants, SB in more than a third and 54% reporting weight gain.

The present study indicates that it has been challenging for individuals during the home confinement period to adequately maintain their regular PA patterns (only 20%). Fifty-two percent (52%) was performing occasional PA and 28% remained completely inactive. Routine disturbance and lack of supportive environment were the most frequently reported reasons. A similar pattern of findings was evident around the globe in both children and adults.^24–26^ COVID-19 studies have showed a decrease in PA and a concomitant increase in SB, including ST.^27–29^ People who were physically active prior to the COVID-19 restriction were more likely to sustain their PA practice.^30^

### Sitting Time

In this study, the reported mean sitting time per day was 5.1 ± 4.7 hours with 38% exposed to 8 hours or more of sedentary activities. A prospective cohort study showed an increase in sitting time by 275 min/week accompanied by decline in the mean level of PA by 333 METs minutes/week among older adults.^31^ Decrease in the step-count was observed in many populations by as much as 15% in the first 30 days of the lockdown.^5^ A systematic review and meta-analysis showed a positive association between sedentary time and risk of death at levels exceeding 9.5 hours per day.^32^ Association with even lower levels were estimated in another meta-analysis at 6–8 hours/day.^33^

### Screen Time

Our study indicates that, on average, participants spent 4±2.8 hours per day engaging in ST (whether recreational or work-related). A systematic review and meta-analysis, including 89 studies signifies that during the pandemic, 51% of the adults have reported increases in both the total and leisure ST.^34^ In this context, Schmidt et al indicated a correlation between ST and physical inactivity in association with different degrees of restrictions during lockdown.^35^ Higher time spent on TV watching and computer use was correlated with moderate-to-severe depression level (adj OR 2.3, 95% CI: 1.602–3.442) in a study among US adults,^36^

### Sleep Quality

Sleep is also a fundamental element for mental well-being as well as in handling stress and anxiety. In this study, more than half of the participants reported a significant decrease in daily sleeping hours (ie, 58% slept less than 7 hours) and insomnia (ie, 53% woke up early and were unable to go back to sleep). Consistently, Mandelkorn et al found that among 3602 adults from 49 countries, 58% were unsatisfied with their sleep, 40% had decreased sleep quality and sleeping pill consumption increased by 20% during the pandemic.^37^ Other studies have correlated the disturbance in sleep circadian rhythm with decreased sunlight exposure or accelerated stress level.^38^,^39^

### Stress Level

Mental health during the COVID-19 confinement was compromised due to several reasons including restricted social interactions, leisure activities, and an overall limit in time spent outdoors. It was observed in our study that 61% had moderate level of stress, while 5% have high level, with mean PSS score of 16.1± 6.5. Similar findings were reported by several studies confirming higher levels of anxiety, stress, and depression during lockdown.^40^,^41^ In India, Grover et al found that 70% of the participants had moderate level of stress after the onset of the lockdown period with mean PSS score of 16.56 ±5.60.^42^

### Obesity and Sedentary Behavior

Prolonged stay at home may have exacerbated SB and frequent snacking of high caloric food, leading to weight gain, in a similar way observed during lengthy summer vacations. In our study, the mean BMI was 30.5 ± 5 kg/m^2^ with more than half of the participants (57%) classified under the obesity category (42% obesity class I, 11% obesity class II and 4% obesity class III). A systematic review confirms that individuals who were overweight/obese or struggled with weight management prior to the lockdown, the case of our participants, were more likely to experience weight gain.^43^ Furthermore, patients with chronic conditions such as diabetes, hypertension, cardiovascular disease, or mental health illnesses affecting their daily living activities were more liable to weight gain.^44^,^45^ Overweight and obese individuals had a higher 10-year cardiovascular (CVD) risk compared to those with normal weight (9.5% vs 10.1% vs 6.3%, P < 0.001).^46^

In this study, the mean WC was 102 ±12.5 cm, and the mean WHR was 0.92 ± 0.10. WC and WHR are considered more accurate estimates of abdominal obesity, when compared to BMI. They have been shown to be strongly correlated with obesity-related conditions and cardiometabolic risk.^47^ The threshold for increased risks can differ according to gender classified into three categories: low (<94 cm for men, <80 cm for women), moderate (94–102 cm for men, 80–88 cm for women) and high (>102 cm for men, >88 cm for women).^48^ According to the US Department of Health and Human Services, the WHR cutoff points to detect obesity and increased risk of metabolic complications are ≥0.95 and ≥0.80 for males and females,^49^ respectively; ≥1.0 and ≥0.85, according to the WHO.^50^ In Turkey, TAĞRAF et al found that when compared to pre-pandemic period, there has been increased in the high-risk category based on WC in both genders, with higher rates observed in males.^51^

Alternatively, arm, calf, and thigh circumferences are generally used to describe musculature/lean mass. Factors such as aging, PA, nutrition and chronic health conditions can influence these measures. In the present study, the mean right and left MUAC were 33 ± 3.9 cm and 32.5 ± 3.9 cm, respectively. The mean right and left CalF were 39 ± 4.2 cm for both sides. Values less than 34 cm in men and <33 cm in women have been shown to predict low MM.^52^ However, when compared to the average CalF across all ages (53.8 cm for men and 52.9 cm for women), participants in this study have shown lower ranges. Due to lack of baseline data, this finding should be interpreted with caution, as the average thigh circumference tends to decrease with age, and a large portion of our study sample comprised the elderly category. Previous studies have demonstrated an inverse association between CalF and cardiometabolic diseases such as diabetes, dyslipidemia, peripheral vascular disease, and all-cause mortality.^53^

### Body Composition

Our study indicates that the prevalence of body adiposity shows a higher-than-normal average level of FM and PFM with a mean of 34 ± 10.5 kg and 42.7 ± 7.2%, respectively. PFM has been shown to be a better indicator of cardiovascular risk, worse lipid profile and mortality when compared to the traditional used BMI.^54^,^55^ Zeng et al indicated a 3%, 5% and 3% risk increase on hypertension, dyslipidemia, and hyperglycemia per 1% increase on PFM, respectively.^56^ To date, there is no consensus for the definition of obesity based on PFM; however, values >35% for women and >25% for men have been ascertained as cut-off points.^57^ Macek et al indicated that those with PFM exceeding the cut-off points were 2–4 times more likely to develop cardiovascular risk factors when compared to those with PFM within normal limits.^58^

Furthermore, looking at the result of this study, the mean MM and PM were below the normal values (20.6 ± 5.4 kg and 25.4 ± 3.9%, respectively). Burrows et al indicated that low MM has been associated with cardiometabolic risk factors independent of dietary intakes.^59^ In agreement, Heo et al found that MM has significantly decreased in all ages during the pandemic when compared to the pre-pandemic period, particularly among older age (60s) by 24.5%. Evidence indicates that prolonged period of physical inactivity (in the case of COVID-19 lockdown) decreases the size of the muscle fiber and therefore leads to loss of muscle function and quality.^60^

Our study indicates a lower-than-normal value of the mean TBW percentage (40.6 ± 6.4%). Inadequate water intake is correlated with metabolism impairment, diabetes mellitus and body weight management.^61^,^62^ Water intake during the pandemic varied across the literature, in Indonesia, the COVID-19 related lockdown has led to inadequate daily water intake [1882 (147–2433) mL/day] among workers.^62^ Similarly, Al-Domi et al reported that 31.4% of adult people in Jordan did not achieve their adequate water intake (≤8 cups) during the COVID-19 pandemic lockdown.^63^ In comparison, Bailly et al in their study assessed the impact of the lockdown in France on weight loss and body composition modifications in subjects participating in a weight loss program and found that there has been a slight increase in the TBW percentage by 0.2 at pre, 0.6 during and 0.1 after lockdown.^64^ Variations could be related to the data collection tools, comparison to pre-pandemic status, time/duration of lockdown and the population studied.

### Cardiopulmonary and Muscular Fitness

In this study, the mean overall cardiopulmonary fitness as reflected by VO2 max was 15.5 ± 11 mL.kg-1.min-1, a much lower estimate (<5^th^ percentile) than the age and sex-predicted values. The ACSM Guidelines for Exercise Testing and Prescription^65^ identifies those with VO_2_ max below the 20th percentile for their age and sex as having very poor to poor cardiorespiratory fitness. The average VO2max is around 35–40 mL/kg/min for an untrained man and around 27–31 mL/kg/min for an untrained woman.^66^ Considering that there was a significant decline in the level of PA during the pandemic, especially among those who were initially very active, Vivan et al^67^ found that VO2 MAX was dropped by 16.7% among previously active runners within the period of two years during the pandemic, a significantly higher value than that expected due to natural aging (ie 1%).^68^ Likewise, in Korea, a study conducted in an exercise rehabilitation center identified reduction in the cardiopulmonary endurance across all age groups (ranging from 6 to 18%), after the detraining following a one-year home confinement. Similar rates were observed in the best rest studies.^69^

Our study indicates that muscular endurance amongst the participants was below the average for age and sex. Evidence from the literature implies that forced confinement has led to a progressive detraining not only in the cardiopulmonary endurance but also the musculoskeletal parameters (muscle mass, strength and power).^70^,^71^ Resistance training assists in maintaining the basal metabolic rate and reduces the risk of falls in the elderly.^71^ In the current study, the mean push-up indicated by the number of repetitions in 1 minute was 19 ± 8.9 reps, and the mean duration of L-sit wall test was 46 ±35 seconds, and the mean duration of plank test was 42.7 ±27.8 seconds. Evidence suggested that 18% of MM can be lost within 90 days of immobilization^72^ with negative health benefits seen only within 3–14 days of sedentarism.^73^ Furthermore, reduced PA was correlated with an increase in insulin resistance and a decline in the postprandial rate of muscle protein synthesis among previously healthy elderly. Heo et al^60^ demonstrated that detraining of approximately 45 weeks in physically active male has led to MM reduction by 8%, 11%, 10%, 13% and 24% in the 20s, 30s, 40s, 50s and 60s age groups, respectively, while Fox et al^74^ indicated that most of the benefits obtained from physical training, including changes in muscle enzymes activity and function are lost within only 4–8 weeks of detraining. Closure of sports facilities during COVID-19 pandemic has led to an average decrease in muscle endurance by 14.4%.^60^

In summary, despite the negative consequences brought by the pandemic, it has provided opportunities to reconsider lifestyle habits and to work toward improving health-related behaviors especially among those who were physically active. It is critical to enhance awareness of the PA benefits and the recommended guidelines, coupled with affordable and accessible strategies to increase PA such as wearable fitness tracker, purchase home-based exercise equipment, accessing virtual fitness platforms, reduce ST and promote sleep hygiene. A useful tool to bring about behavioral modification is to consider self-monitoring approaches such as goal setting, feedback on performance, and review of behavioral objectives. Designing an effective wellness program that is patient-centered, tailored to clinical condition and needs with continuous monitoring of performance, adherence and outcomes will help individuals return and maintain physical fitness, reduce health-risks, chronic disease, and premature mortality.

## Limitations

The findings of this study ought to be considered in view of a couple of limitations. First, the cross-sectional nature in a solitary Wellness Center does not allow casual inferences in the results and reduced the generalizability of our results. Secondly, despite the use of validated tools, some of the lifestyle behaviors and presence of chronic diseases were based on self-reports and could have been subjected to self-selection and reporting bias. Thirdly, lifestyle changes were assessed after two years of service closure and thus may be susceptible to recall bias. Fourthly, in this study, we have not compared the PA and lifestyle behaviors, anthropometric measures, body compositions, cardiopulmonary or muscular endurance before and after the pandemic, which could have limited drawing inferences and conclusive remarks. Fifthly, the convenience sampling method led to overrepresentation of female (ratio of male:female at wellness is 1:3), well-educated and married participants who were possibly more health conscious. Nonetheless, there was reasonably good representation of ages and income groups. Lastly, PA and stress level were assessed using validated tools; however, the timespan of measure is 7 days and one month, respectively, and given the fact that the beginning of the pandemic appears to have had the greatest impact on mental health and our study measures these changes after two years where other factors like vaccine availability, reduced mortality, lifted restrictions and people adjustment may have allowed them to deal with stress better and underestimated reporting.

## Conclusion

Physically active adults have experienced negative lifestyle behaviors during the COVID-19 lockdown. Obesity, unfavorable body composition, low cardiopulmonary and muscular endurance were evident following the two years wellness facility closure. This finding calls for practitioners and health policy makers to develop targeted intervention strategies to promote healthy lifestyle during any potential future crisis.

## Implication on Practice and Research

The present study suggests that the new changes brought by the pandemic have brought about negative changes in lifestyle behavior leading to increased obesity, SB, sleep, and stress disorders. These findings provide a viewpoint about the potential forthcoming increase in noncommunicable disease burden and will aid healthcare professionals to better identify priorities and reevaluate cardiovascular as well as metabolic risk profile, engage people in appropriate lifestyle counselling and adaptive skills. The adverse changes in the lifestyle habits seen in our study population (physically active clients) would have probably been more pronounced in the general, inactive population. Future emergencies or lockdowns necessitate the early plan of promoting regular at home exercise and working on mental health and sleep hygiene as early mitigation strategies. In Wellness Center, the use of e-platforms is recommended for the delivery of these interventions to help wellness clients to adopt sustainable changes for future possible service closure.

Longitudinal studies with representative samples should be conducted to better understand the long-term effects of the COVID-19 lockdown and the restrictions put in place on lifestyle habits, their changes over time and association with the resumption of wellness exercise program.

## Data Sharing Statement

The data presented in this study are available on request from the corresponding author. The data are not publicly available due to the privacy of participants.
